# Influence of Ionic Strength and Temperature on the Adsorption of Reactive Black 5 Dye by Activated Carbon: Kinetics, Mechanisms and Thermodynamics

**DOI:** 10.3390/molecules30122593

**Published:** 2025-06-14

**Authors:** Mario Cetina, Petra Mihovilović, Ana Pešić, Branka Vojnović

**Affiliations:** Department of Applied Chemistry, Faculty of Textile Technology, University of Zagreb, Prilaz Baruna Filipovića 28a, HR-10000 Zagreb, Croatia; petra.mihovilovic@ttf.unizg.hr (P.M.); branka.vojnovic@ttf.unizg.hr (B.V.)

**Keywords:** isothermal adsorption, activated carbon, Reactive Black 5, ionic strength, kinetics, thermodynamics

## Abstract

The aim of this work was to investigate the influence of ionic strength and temperature on the adsorption of Reactive Black 5 dye on commercial powdered activated carbon. Adsorption experiments were performed at 45 °C with the addition of NaCl (*c*_0_ = 0.01, 0.05, 0.10 and 1.00 M) and Na_2_SO_4_ (*c*_0_ = 0.01 M). The results were compared with those obtained for both salts (*c*_0_ = 0.01 M) at three additional temperatures: 25, 35 and 55 °C. For all adsorption experiments, kinetic and thermodynamic studies were performed. This research showed that the addition of NaCl, even in the concentration of only *c*_0_ = 0.01 M, significantly enhanced dye adsorption and that higher NaCl concentration resulted in higher adsorption capacity. In addition, slightly higher adsorption was observed when Na_2_SO_4_ was added to the dye solution at the same concentration as NaCl, as well as at a higher temperature, regardless of the salt added to the dye solution. It was also shown that adsorption is kinetically controlled, assuming a pseudo-second-order model, and that intraparticle diffusion is not the only process that influences the adsorption rate. Finally, calculated thermodynamic parameter values for both salts (*c*_0_ = 0.01 M) indicate that adsorption was a spontaneous endothermic process.

## 1. Introduction

The textile industry, as one of the most resource-intensive industries, produces large amounts of wastewater. Textile wastewaters are characterised by altered colouration and significantly increased values of temperatures, pH, conductivity, turbidity and suspended matter accompanied by high chemical oxygen demand (COD) and biochemical oxygen demand (BOD) [1]. The dyeing process is one of the most important steps in textile materials production. According to an estimate from 2019, approximately up to 280 tonnes of textile dyes are discharged yearly as industrial effluent [2], and more than 10% of excess dyestuff is being discharged into water bodies due to incomplete dye exhaustion [3]. Even a low amount of dye present in water is noticeable as a significant change in colour, which is aesthetically unpleasing and ecologically unacceptable. Dyes present in waters inhibit light penetration, oxygen production and consummation, enter into the food chain, bioaccumulate, disrupt photosynthesis and exhibit a potential to induce mutagenic and carcinogenic effects [4]. In addition, some dyeing process involves the usage of different auxiliaries, such as carriers and inorganic salts, or the dye itself contains metal ions [5].

Synthetic dyes are usually classified into the following groups according to their chemical structure and mode of application: anionic-direct, acid and reactive dyes, cationic-basic dyes, and non-ionic-disperse dyes. Reactive dyes, which usually exhibit vibrant colours, are typically azo-based chromophores combined with different types of reactive groups, such as vinyl sulfone, chlorotriazine, trichloropyrimidine and difluorochloropyrimidine. They are among the most widely used dyes, accounting for at least 70% of all dyes used. The removal or mineralisation of water-soluble reactive dyes is extremely challenging. As they are electron deficient, they are less susceptible to degradation in the presence of light, heat, oxidant agents and microorganisms. Consequently, they tend to pass through conventional treatment systems unaffected. In addition, the cleavage of azo bonds can lead to the formation of toxic amines in the effluent [6,7]. Having that in mind, such wastewater poses a serious threat to the environment if it is not appropriately treated before discharge.

Various methods and technologies have been used for decolourisation of textile wastewater with varying efficacies. Available methods are generally divided into physico-chemicals processes, such as coagulation and flocculation [8]; adsorption and membrane technologies [9,10]; biological processes, including microbial or enzymatic digestion [11]; and advanced oxidation processes [12]. Each of the previously mentioned decolourisation methods has some benefits and shortcomings. A single technique might not be enough to accomplish total decolourisation of wastewater, so there may be a need to use a combination of methods. Biological and chemical oxidation methods are the most frequently used treatment processes in municipal wastewater treatment plants as they have the ability to completely degrade the toxic substances and their by-products. For coloured textile wastewater membrane technologies, and to a lesser extent, coagulation/flocculation is also applicable [13,14].

Adsorption stands out among other methods in the first place for its great decolourisation efficiency of wastewater containing a variety of dyes. It is also economically suitable and eco-friendly; it has the simplest operation, as well as recyclability of the adsorbents. Recent studies on the application of different adsorbent materials for the removal of reactive dyes provide a wide variety of different types of available adsorbents such as commercial activated carbon, metal oxide-based, carbon-based, metal–organic frameworks, polymer-based adsorbents [15] and non-conventional bio-adsorbent materials obtained from agricultural waste (e.g., coffee ground powder or pine tree leaves) [16,17]. Because of their large surface area, nanosize, high reactivity, and ability to blend, modern adsorbent materials show promise in a variety of applications.

**Figure 1:**
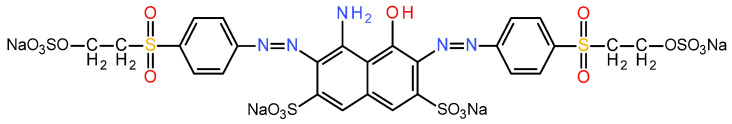
Chemical structure of Reactive Black 5 dye.

Activated carbon is still one of the most popular adsorbents used for wastewater treatment due to its low costs, easy accessibility, great adsorption efficiency and wide applicability ([18], and references cited therein). It is well known that adsorption is a time-dependent process, influenced by many factors such as temperature, the optimal dosage of the adsorbent, the initial concentration of the present dye and the pH value of the solution. Our previous papers primarily focused on the influence of initial dye concentration and pH value on the adsorption of Reactive Black 5 (RB5) dye onto commercial powdered activated carbon [19,20]. As different salts are used during the dyeing processes and ionic strength also plays a crucial role in the dye adsorption process, in this paper, we report the influence of two salts on the adsorption of RB5 dye (Figure 1) at 45 °C on the same adsorbent. Compared with other similar studies (vide infra), the novelty of the present work lies in the usage of sodium sulphate (*c*_0_ = 0.01 mol dm^−3^) in exchange for sodium chloride (*c*_0_ = 0.01, 0.05, 0.10 and 1.00 mol dm^−3^), both serving as neutral salts. Due to its influence on the adsorption process, pH value was also monitored. Furthermore, as temperature dependence on the adsorption process is also of great importance and wastewater temperatures can vary in a wide range, we also performed adsorption experiments at 25, 35 and 55 °C for salts concentration of *c*_0_ = 0.01 mol dm^−3^. Finally, kinetic and thermodynamic studies for these adsorption systems were also performed.

## 2. Results and Discussion

### 2.1. Adsorbent Characterisation

In order to characterise activated carbon, we first measured its specific surface area, and it was determined to be 814.8 ± 25.8 m^2^ g^−1^.

FTIR spectra analysis was also used to characterise activated carbon and identify its surface functional groups. FTIR spectrum of activated carbon used in adsorption experiments displays several characteristic bands associated with functional groups present in activated carbon (Figure S1, Supplementary Material). The band observed at 3449 cm^−1^ corresponds to O–H stretching vibrations and indicates the presence of alcoholic, phenolic, and carboxylic groups on the activated carbon surface. Multiple bands around 2900 cm^−1^ are related to the symmetric and asymmetric stretching vibration of aliphatic C–H in present methyl, methylene or methine carbon atoms. The band at 2368 cm^−1^ is attributed to C–H stretching due to the presence of the CH_2_–CO group. The peak at 1629 cm^−1^, characteristic of C=O stretching, suggests the presence of carbonyl-containing functional groups such as ketones, carboxylic acids, esters or lactones. Additionally, combination bands in the region between 1550 and 1400 cm^−1^ confirm the presence of aromatic rings in the structure. Finally, multiple bands in the 1300–1000 cm^−1^ range indicate the presence of other oxygen-containing groups in activated carbon, including anhydrides, lactones, esters, ethers, or C–O stretching in combination with O–H bending mode in phenol and carboxylic acids. These bands may also overlap with those from C–H aliphatic and aromatic deformation vibrations.

**Figure 2:**
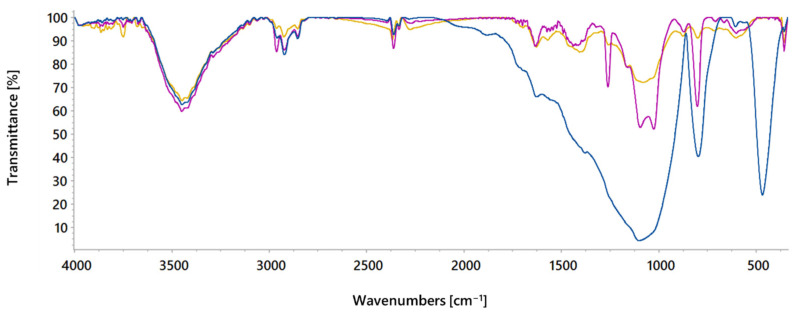
FTIR spectra of activated carbon before (**—**) and after treatment with 0.01 M NaCl (**—**) and 0.01 M Na_2_SO_4_ (**—**).

To investigate the effect of sodium chloride and sodium sulphate on activated carbon surface functional groups, FTIR spectra were also recorded for activated carbon treated with 0.01 M salt solutions (Figure 2). Compared to the untreated activated carbon, the treated samples exhibited stronger spectral bands, which may be attributed to electrostatic interactions with the electrolytes that enhance bond dipole moments and increase IR absorption. Notably, significant changes, particularly below 1300 cm^−1^, associated with C–O stretching, suggest interactions between Na^+^ ions and oxygen atoms. Additionally, the appearance of a new band at 802 cm^−1^ may result from interactions between Cl^−^ ions and hydrogen atoms in carboxylic groups. A similar effect is observed in activated carbon treated with Na_2_SO_4_. However, a prominent band around 1100 cm^−1^ that appears could be attributed to the S=O stretching vibration of SO_4_^2−^ ions bonded to the activated carbon surface.

**Figure 3:**
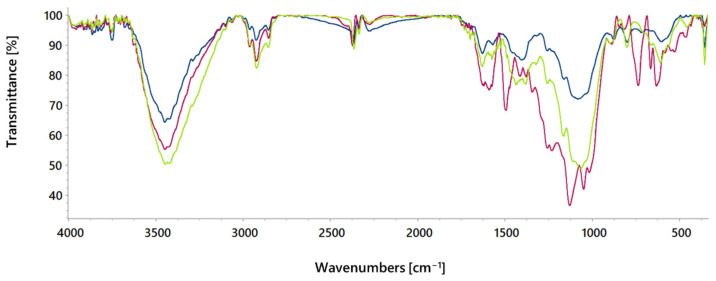
FTIR spectra of activated carbon before (**—**) and after RB5 dye adsorption (**—**), as well as of RB5 dye (**—**).

To assess the adsorption mechanism of the RB5 dye on the activated carbon, the FTIR spectrum of activated carbon is also compared with that recorded after RB5 dye adsorption, alongside the spectrum of the RB5 dye itself (Figure 3). Previous characterisation confirmed that activated carbon surface is rich in acidic functional groups, which serve as binding sites for reactive dye anions. After RB5 adsorption, the –OH band shifts to lower wavenumbers with increased intensity, suggesting hydrogen bond formation between the hydroxyl groups of activated carbon and the dye. Similar shifts are observed in the C–O stretching bands (ṽ = 1064, 1169, and 1258 cm^−1^), indicating interactions involving oxygen atoms. The shift of the C=C vibration band, along with the disappearance of deformation bands of =C–H bond in the fingerprint region (δ = 700–1100 cm^−1^), suggest the involvement of activated carbon’s aromatic rings in π–π interactions. This is further supported by the disappearance of bands at 1592 and 1494 cm^−1^ in the RB5 spectrum, confirming the π–π interactions between the dye’s conjugated and activated carbon aromatic rings. Finally, the loss of S=O and C–S bands indicate the involvement of the SO_3_^−^ RB5 dye groups in surface interactions.

**Figure 4:**
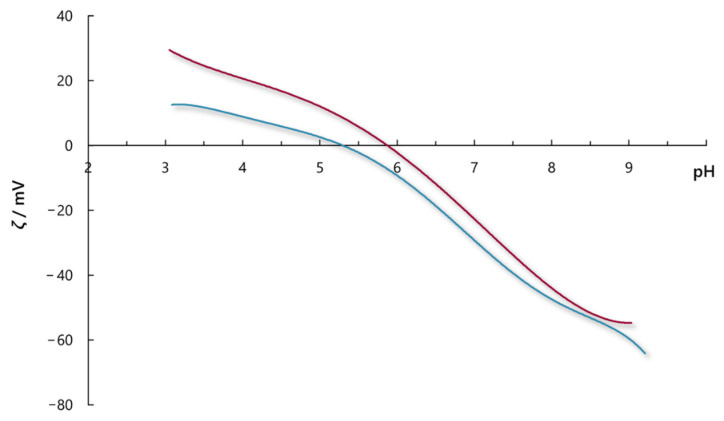
Electrokinetic ζ-potential of the activated carbon in sodium chloride (**—**) and sodium sulphate (**—**) aqueous solutions as a function of pH value.

Zeta-potential measurements of activated carbon obtained with the addition of sodium chloride and sodium sulphate (*c* = 10^−3^ mol dm^−3^) are presented in Figure 4. The potential where the charge on the surface is zero is called an isoelectric point (IEP), and the corresponding pH is designated as pH_iep_. Under conditions when pH < pH_iep_-activated carbon has a positive surface charge, while at pH > pH_iep_, the surface charge becomes negative.

Figure 4 shows that the pH_iep_ for activated carbon aqueous solution determined with the addition of sodium chloride is at pH ≈ 5.8, while for sodium sulphate, it is at pH ≈ 5.2. It is known that an electric double layer formed on the charged surface depends on electrolyte concentration and the charge of the ions. The slightly lower value of the pH_iep_ obtained with the addition of sodium sulphate can be explained by the fact that an increase in ion charge (i.e., in the case of SO_4_^2−^ ions) for equivalent solution concentrations, compresses the double layer, leading to a lowering of pH_iep_ [21].

**Figure 5:**
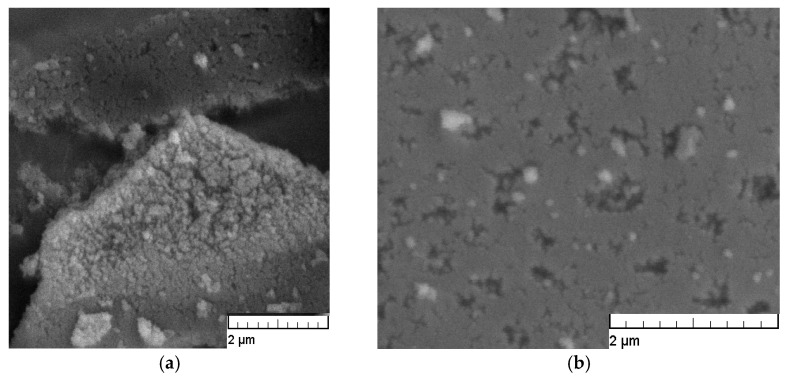
SEM images of activated carbon surface enlarged: (**a**) 20,000× and (**b**) 30,000×.

Finally, in order to carry out a morphological analysis of activated carbon, photographs were taken using scanning electron microscopy (SEM). In recorded photographs of activated carbon, it can be seen that the particles differ morphologically and that they have different pore sizes (Figure 5).

### 2.2. Effect of Ionic Strength and Temperature on Adsorption Process

As wastewater from the dyeing process contains a high concentration of electrolytes, it is very important to examine the effect of ionic strength on the dye adsorption. It is known that dyeing of 1 kg of cotton with reactive dyes requires 30–60 g of dyestuff, 70–150 L of water and 600 to 800 g of sodium chloride, i.e., dyeing is performed with NaCl in concentrations ranging from ca. 0.01 to 0.2 mol dm^−3^ depending on the dyeing bath volume [22]. For the adsorption process was chosen Reactive Black 5 (RB5) dye in the concentration of *c*_0_ = 500 mg dm^−3^, as many textile industry effluents contain approximately such concentration of dye after dyeing process. The first aim of this work was to examine the influence of sodium chloride concentration on RB5 dye adsorption on activated carbon at the temperature of 45 °C. For that objective, sodium chloride was added to the dye solution in four initial concentrations: *c*_0_ = 0.01, 0.05, 0.10 and 1.00 mol dm^−3^ (in further text designated as M). Table 1 and Figure S2 (Supplementary Material) show amounts of adsorbed dye (*q*_t_) during the adsorption process from 15 min to 16 h, when equilibrium was reached, for different NaCl concentrations, as well as results obtained without salt addition during the adsorption process [23].

**Figure 6:**
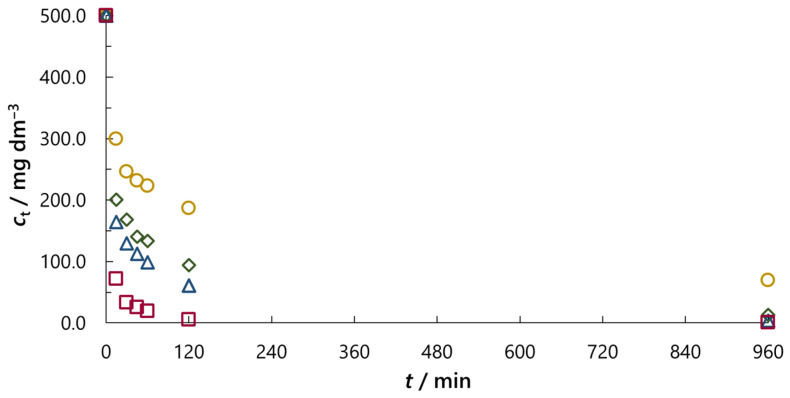
Effect of NaCl concentration on the RB5 dye concentration in the liquid phase (*c*_t_) after appropriate time of adsorption (*t*) at 45 °C (**◯** *c*_0_ = 0.01 M; **◇** *c*_0_ = 0.05 M; **Δ** *c*_0_ = 0.10 M; **☐** *c*_0_ = 1.00 M).

Data in Table 1 show that the addition of sodium chloride in RB5 dye solution significantly enhanced dye adsorption. Even the addition of NaCl in the concentration of *c*_0_ = 0.01 M increased the amount of adsorbed dye on the activated carbon after 15 min for ca. 37 mg g^−1^ (for ca. 58%). Furthermore, after 30 min for *c*_0_(NaCl) = 0.05 M, the amount of adsorbed dye is almost twice as big compared to the adsorption process when salt is not added (166.0 vs. 83.6 mg g^−1^, Table 1). As expected, the highest adsorption capacity, i.e., the amount of adsorbed dye at equilibrium (*q*_e_), was observed for *c*_0_(NaCl) = 1.00 M (249.9 mg g^−1^). However, an increase in adsorption capacity with an increase in NaCl concentration is not proportional. The adsorption capacity for NaCl concentration of *c*_0_ = 0.01, 0.10 and 1.00 M (10 times range) was ca. 215, 248 and 250 mg g^−1^, which corresponds to 1.13, 1.30 and 1.31 times increase compared to adsorption without NaCl (ca. 191 mg g^−1^). This also means that the difference between amounts of adsorbed RB5 dye (*q*_t_) for different NaCl concentrations are at the beginning of adsorption bigger in comparison with those at later stages of adsorption. The influence of different NaCl concentrations on the adsorption is more clearly evident in Figure 6, where liquid phase dye concentration (*c*_t_) during the adsorption process is presented.

Figure 6 shows that RB5 dye concentration in the liquid phase decreased quickly for all NaCl concentrations. However, adsorbate concentration for *c*_0_ (NaCl) = 1.00 M fell down extremely rapidly, and after 15 min is already less than 100 mg dm^−3^. For other NaCl concentrations (from *c*_0_ = 0.01 to 0.10 M), the curves can be approximately divided into three regions: initial very fast adsorption (up to 45 min); milder adsorption; and gradual decrease in dye concentration, which reaches the equilibrium state after 16 h. In order to examine how some other salts affect adsorption, we investigated four sodium chloride concentrations and found the smallest one to be optimal (*c*_0_ = 0.01 M). Therefore, we performed adsorption experiments with another neutral salt used in dyeing processes, sodium sulphate, applying the same concentration (*c*_0_ = 0.01 M) and conditions (dye concentration, mass of adsorbent and temperature). Calculated efficiency of decolouration (*E*_d_, Equation (10)) obtained for dye solutions with these salts added, as well as without salt addition during the adsorption process [23], are given in Table 2.

**Figure 7:**
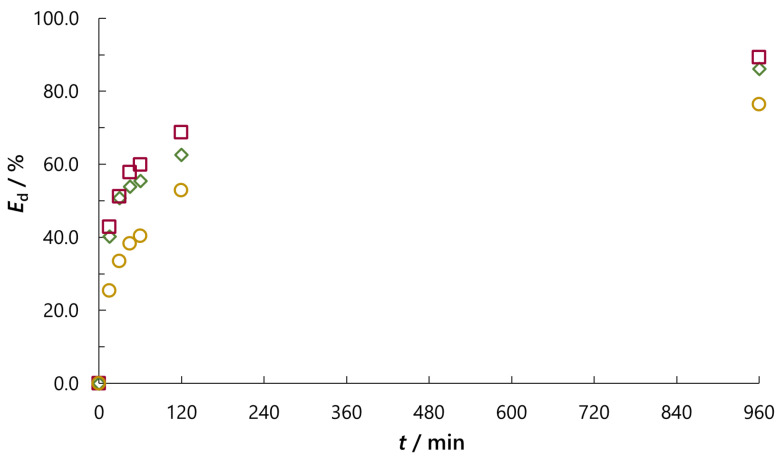
Effect of ionic strength (*c*_0_ = 0.01 M) on the efficiency of decolouration (*E*_d_) after appropriate time of adsorption (*t*) at 45 °C (**◯** without salt added; **◇** NaCl; **☐** Na_2_SO_4_).

Data in Table 2 clearly show the influence of salt type on the adsorption process. As previously described, the addition of NaCl, even at such a small concentration (*c*_0_ = 0.01 M), significantly improves adsorption. The efficiency of decolouration (*E*_d_) after 30 min is for ca. 17% higher with the addition of NaCl than without it, while after 60 min, *E*_d_ is higher for ca. 15%. Also, due to ionic strength, for the same adsorption periods, *E*_d_ in the case of Na_2_SO_4_ (*c*_0_ = 0.01 M) is continuously higher. Namely, the ionic strength equation, besides concentration, emphasises the charges of the ions because the charge numbers occur as their squares. For all NaCl concentrations (*c*_0_ = 0.01, 0.05, 0.10 and 1.00 M), ionic strength is approximately equal to the concentration (*I* ≈ 0.01, 0.05, 0.10 and 1.00), whereas for Na_2_SO_4_ (*c*_0_ = 0.01 M) due to the number of sodium cations that arises by its dissociation and charge of the sulphate anion *I* ≈ 0.03. However, the differences in *E*_d_ between sodium sulphate and chloride of the same concentration are not so significant, the largest being ca. 6% for adsorption time of 2 h (Figure 7 and Table 2).

The results proved that higher ionic strength, i.e., higher NaCl concentration or number of present ions in solution and their charge in the case of Na_2_SO_4_, improves RB5 dye removal (Table S1, Supplementary Material). Thus, for all experiments performed at 45 °C adsorption follows the sequence *E*_d_ [*c*_0_ (NaCl) = 1.00 M] > *E*_d_ [*c*_0_ (NaCl) = 0.10 M] > *E*_d_ [*c*_0_ (NaCl) = 0.05 M] > *E*_d_ [*c*_0_ (Na_2_SO_4_) = 0.01 M] > *E*_d_ [*c*_0_ (NaCl) = 0.01 M].

Just a few papers dealt with the influence of ionic strength on the adsorption of Reactive Black 5 onto carbon [24,25,26,27,28]. In all of them, the presence of sodium chloride in an aqueous solution resulted in enhanced adsorption of dye molecules on the carbon surface. Along with sodium chloride, only one study used sodium phosphate, yielding the same result, i.e., enhanced adsorption compared to results obtained without salt addition [24]. However, it is well known that pH value is one of the most important parameters affecting the adsorption process ([20], and references cited therein). As sodium phosphate solution is not neutral, the adsorption results for NaCl and Na_3_PO_4_ solutions of the same concentration cannot be compared, unlike the results obtained for NaCl and Na_2_SO_4_.

**Figure 8:**
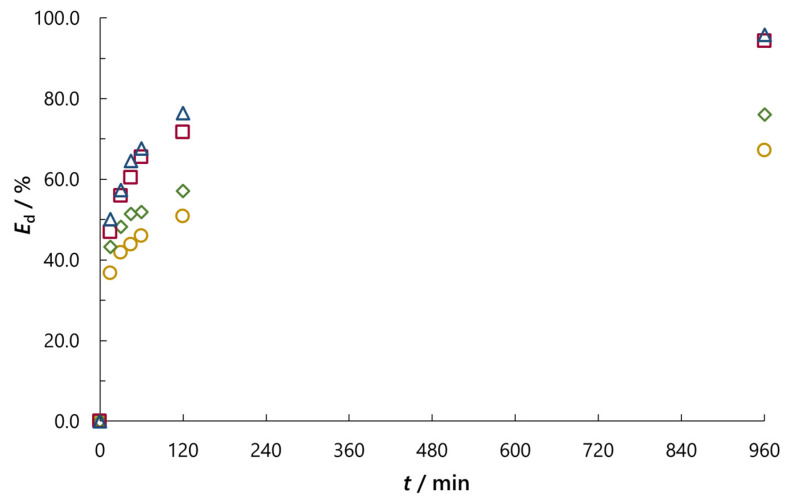
Effect of temperature on the efficiency of decolouration (*E*_d_) after appropriate time of adsorption (*t*) for NaCl and Na_2_SO_4_ concentration of *c*_0_ = 0.01 M (**◯** NaCl, 25 °C; **◇** Na_2_SO_4_, 25 °C; **☐** NaCl, 55 °C; Δ Na_2_SO_4_, 55 °C).

In order to examine the influence of temperature on RB5 dye adsorption, we performed additional experiments at temperatures of 25, 35 and 55 °C with the addition of NaCl and Na_2_SO_4_ (*c*_0_ = 0.01 M) using the same experimental procedure. For comparison, the results of the efficiency of decolouration (*E*_d_) for both salts at all four temperatures are given in Table S2 (Supplementary Material). The data show that at each temperature, during all adsorption periods, the addition of Na_2_SO_4_ resulted in higher adsorption of RB5 dye. In addition, higher temperatures caused greater adsorption, regardless of the salt added to the dye solution. Figure 8 illustrates the differences in *E*_d_ for the addition of NaCl and Na_2_SO_4_ at the lowest and highest temperatures, 25 and 55 °C. The differences in *E*_d_ between these two salts for all four temperatures range from 0.3% (after 30 min at 45 °C) to 8.9% (after 16 h at 25 °C; Table S2, Supplementary Material).

**Figure 9:**
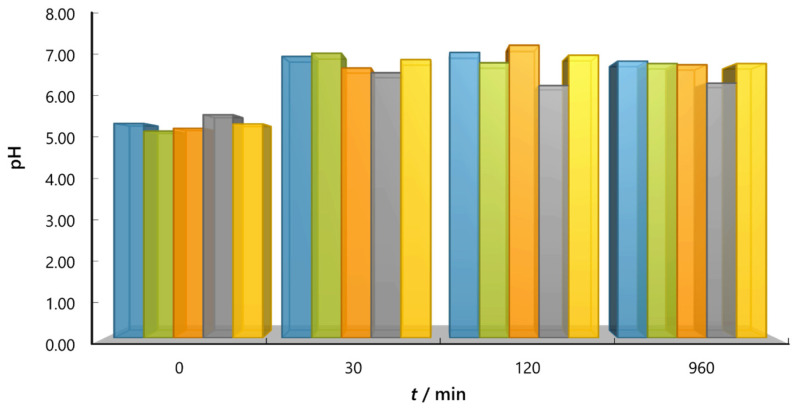
pH profiles for RB5 dye adsorption at 45 °C at the beginning of adsorption, after 30 min, 2 h and 16 h for all NaCl concentrations (■ *c*_0_ = 0.01 M; ■ *c*_0_ = 0.05 M; ■ *c*_0_ = 0.10 M; ■ *c*_0_ = 1.00 M) and Na_2_SO_4_ concentration of *c*_0_ = 0.01 M (■).

The additional aim of this work was to prove that although both salts are neutral, their addition to the dye solution does not influence the pH value during the adsorption process and consequently on adsorption intensity. It was found that neither NaCl nor Na_2_SO_4_ significantly influenced the pH value despite salt concentration and temperature (Figure 9, Figure S3 and Table S3 in Supplementary Materials). It is known that besides RB5 dye, the surface of activated carbon adsorbs more H^+^ ions than OH^−^ ions [29], as also determined in this study by the zeta potential measurements [pH_iep_(NaCl) ≈ 5.8, pH_iep_(Na_2_SO_4_) ≈ 5.2], i.e., the surface of activated carbon from pH_iep_ value to pH = 7 is negatively charged (Figure 4). Therefore, the pH values at the beginning of adsorption increase and, after some time, gradually form a plateau and stabilise at a pH value of ca. 6.5–7.0. Thus, the addition of salts into the RB5 dye solution did not significantly affect adsorption, i.e., the efficiency of decolouration, during the whole adsorption time.

Based on the results of zeta potential and FTIR spectra analysis, the adsorption mechanism of RB5 dye on the activated carbon surface can be proposed. pH values of the dye solutions after adsorption are throughout the adsorption time bigger than pH_IEP_ despite the salt used in the adsorption experiment (Figure S3, Supplementary Material). This indicates that the activated carbon surface during the adsorption process is negatively charged and that electrostatic interactions are not the driving force for the adsorption process because both the dye anion and the activated carbon surface carry the same charge. Based on FTIR spectra analysis it can be concluded that salt cations (Na^+^ ions) serve as a bridge between activated carbon groups and dye anions. This means that electrostatic interactions, in fact, link the activated carbon surface and cations, which in turn interact with dye anions. These interactions could be a reason why the addition of salts, even at very low concentrations, significantly enhances the adsorption of RB5 dye onto commercial powdered activated carbon (Table 2). Slightly greater adsorption obtained by the addition of Na_2_SO_4_ could be explained by a doubled number of cations involved in these interactions. Of course, in addition to electrostatic, many other interactions contribute to the dye adsorption process on the activated carbon, primarily hydrogen bonds, which were also observed in FTIR spectra. Furthermore, interactions involving activated carbon or/and dye aromatic rings are also present. This includes cation–π interactions between sodium ions and activated carbon aromatic rings, anion–π interactions between chloride or sulphate anions and aromatic rings, and π–π interactions between activated carbon and dye aromatic rings, all of which were observed in FTIR spectra. However, increased adsorption after salt addition could also be ascribed to an enhanced dimerisation of dye molecules in solution through several intermolecular interactions that are intensified upon salt addition to the dye solution [30]. Accordingly, an additional reason for the enhanced adsorption of RB5 dye with higher ionic strength can be attributed to the aggregation of dye molecules itself or induced by ions in the dye solution [24,25,26,27].

### 2.3. Kinetics of Adsorption

Experimental data were analysed by pseudo-first-order and pseudo-second-order models, which are widely employed to determine kinetic parameters, as well as by the intraparticle diffusion model.

#### 2.3.1. Pseudo-First-Order and Pseudo-Second-Order Kinetic Models

The linearised forms of the pseudo-first-order [31] and pseudo-second-order [32,33] models are given in Equations (1) and (2), respectively:

(1) $$\ln\left(q_{e}-q_{t}\right)=\ln q_{e}-k_{1}\cdot t$$

(2) $$\frac{t}{q_{t}}=\frac{1}{k_{2\;}\cdot q_{e}^{2}}+\frac{1}{q_{e}}\cdot t,$$

where *k*_1_ (min^−1^) and *k*_2_ (g mg^−1^ min^−1^) are the rate constants of pseudo-first-order and pseudo-second-order, respectively. The pseudo-first-order kinetic constant (*k*_1_) and calculated amount of adsorbed dye at equilibrium (calculated adsorption capacity, *q*_e,calc._) can be determined from the slope and intercept, respectively, by plotting ln(*q*_e_ − *q*_t_) vs. time (*t*). Similarly, the pseudo-second-order kinetic constant (*k*_2_) and *q*_e,calc._ can be obtained by plotting *t*/*q*_t_ vs. time (*t*). This plot enables calculation of *q*_e,calc._ from the slope and the pseudo-second-order kinetic constant (*k*_2_) from the intercept. A linear relationship with a high value of correlation coefficients (*R*^2^) suggests the applicability of Equations (1) and (2). Finally, the initial adsorption rate *h* (mg g^−1^ min^−1^) for the pseudo-second-order kinetic model was calculated using the following equation [33,34]:

(3) $$h=\;k_{2}\cdot q_{e,\mathrm{calc}.\;}^{2}.$$

Kinetic parameters for both models, derived for all sodium chloride concentrations and sodium sulphate concentration of *c*_0_ = 0.01 M at 45 °C, are given in Table 3, while linearised plots for pseudo-first-order and pseudo-second-order kinetic models are depicted in Figures S4–S6 (Supplementary Material).

Data in Table 3 show that the correlation coefficient values (*R*^2^) for the pseudo-first-order model are slightly lower for *c*_0_(NaCl) = 0.01 M (89.6%), while for the other NaCl concentrations and for Na_2_SO_4_ (*c*_0_ = 0.01 M), they are relatively high, ranging from 95.2% to 98.4%. However, there is a great disagreement between experimental and calculated values of the amount of adsorbed dye at equilibrium. Specifically, calculated values (*q*_e,calc._) are from 1.9 to 6.2 times lower than the corresponding experimental values (*q*_e,exp._). On the other hand, the calculated adsorption capacities (*q*_e,calc._) for the pseudo-second-order model approximately correspond to the experimental ones, accompanied by high correlation coefficient values of nearly 100% (Table 3). This indicates that the adsorption of RB5 dye on commercial activated carbon with the addition of both salts, regardless of NaCl concentration, is kinetically controlled assuming a pseudo-second-order process. The pseudo-second-order model considers chemical adsorption as the rate-limiting process. It is also obvious that an increase in NaCl concentration leads to higher values of rate constant (*k*_2_) and initial adsorption rate (*h*), so the highest value was calculated for the maximum NaCl concentration (*c*_0_ = 1.00 M). Also, the rate constant *k*_2_ for Na_2_SO_4_ concentration of *c*_0_ = 0.01 M is slightly higher than that for NaCl of the same concentration (Table 3). As previously mentioned, we also performed adsorption experiments with both salts concentration of *c*_0_ = 0.01 M at three additional temperatures. Table S4 (Supplementary Material) summarises kinetic parameters for pseudo-first-order and pseudo-second-order models for RB5 dye adsorption with the addition of NaCl and Na_2_SO_4_ at 25, 35 and 55 °C. The pseudo-second-order process also kinetically controls these adsorption processes, as evident from the correlation coefficient values and calculated adsorption capacities, which correspond to experimental values.

#### 2.3.2. Intraparticle Diffusion Model

To evaluate the diffusion mechanism, we applied the intraparticle diffusion model. Adsorption processes usually include three sequential stages:

1. Mass transfer of the adsorbate from the bulk solution to the adsorbent surface (film diffusion);
2. Adsorbate adsorption at active sites on the adsorbent;
3. Intraparticle diffusion of the adsorbate into the adsorbent’s pores, followed by adsorption at internal sites.

The second stage (surface adsorption) is typically very rapid and rarely considered as the rate-limiting step. Therefore, it is proposed that the rate of adsorption is governed by the intraparticle diffusion in the pore of the adsorbent. The intraparticle diffusion model is based on the following equation [29,34]:

(4) $$q_{t}=k_{i}\cdot t^{0.5},$$

where *k*_i_ is the intraparticle diffusion rate constant (mg g^−1^ min^−0.5^).

If intraparticle diffusion governs the adsorption rate, a plot of the adsorbed amount of dye (*q*ₜ) vs. square root of the time (*t*^1/2^) should exhibit linearity and pass through the origin. Multi-linearity in this plot indicates that intraparticle diffusion is not the sole rate-limiting step, suggesting the involvement of additional rate-controlling mechanisms during the adsorption process [29,35].

**Figure 10:**
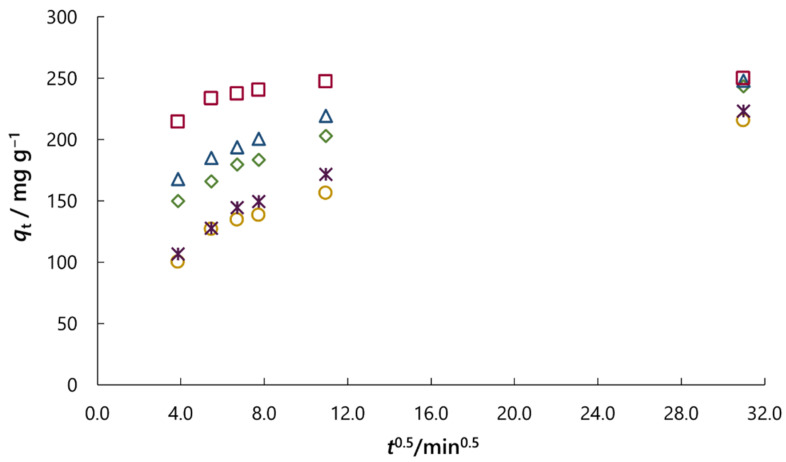
Root time plot for the adsorption of RB5 dye on activated carbon at 45 °C for all NaCl concentrations (**◯** *c*_0_ = 0.01 M; **◇** *c*_0_ = 0.05 M; **Δ** *c*_0_ = 0.10 M; **☐** *c*_0_ = 1.00 M) and Na_2_SO_4_ concentration of *c*_0_ = 0.01 M (✴).

Figure 10 shows the root time plots for the adsorption of RB5 onto activated carbon at 45 °C for all NaCl concentrations and Na_2_SO_4_ concentration of *c*_0_ = 0.01 M. All plots on this figure exhibit multi-linearity, thus indicating that intraparticle diffusion plays an important role during adsorption, but is not the only rate-limiting step. A few stages in several regions can be distinguished during the dye adsorption. The adsorption rate is initially higher and corresponds to instantaneous adsorption (from the beginning of adsorption to 15 min). The amount of adsorbed dye on the adsorbent surface, and thus diffusion decreases over time (from 15 to 45 min and 45 min to 2 h), which represents a gradual adsorption stage where diffusion rates decrease by increasing the contact time due to the intraparticle diffusion of the molecules into the pores of the activated carbon. This stage is followed by the equilibrium stage, during which dye molecules occupy all active sites of the adsorbent (from 2 to 16 h). Diffusion in the pores of the adsorbent is usually determined by the fact that there are several different pore sizes in the adsorbent (see Figure 5) and that the pores for diffusion become smaller, and thus, the free path for the molecules in the pore decreases after the dye molecules diffuse into the inner structure of the activated carbon. Thus, these results show that some other mechanism, along with intraparticle diffusion, influences the adsorption rate and that multiple steps took place during the adsorption process. The same case is with NaCl and Na_2_SO_4_ concentration of *c*_0_ = 0.01 M at 25, 35 and 55 °C (Figures S7 and S8 in Supplementary Materials).

### 2.4. Adsorption Thermodynamics

As previously mentioned, adsorption experiments were performed until equilibrium, with the addition of both salts of the same concentration (*c*_0_ = 0.01 M) at four temperatures (25, 35, 45 and 55 °C). Based on the results obtained, we were able to calculate thermodynamic adsorption parameters: standard Gibbs free energy change values (Δ*G*^o^, kJ mol^−1^), standard enthalpy change values (Δ*H*^o^, kJ mol^−1^), and standard entropy change values (Δ*S*^o^, J mol^−1^). Standard Gibbs free energy change values can be calculated from equation [36]:

(5) $$\Delta G^{o}=-R\;\cdot \;T\;\cdot \;\ln\;(K_{c}),$$

where *R* is the ideal gas constant (*R* = 8.314 J mol^−1^ K^−1^), and *T* is temperature (K). The equilibrium constant (*K*_c_) could be calculated from the initial dye concentration (*c*_0_ = 500 mg dm^−3^) and the concentration of dye in the liquid phase at equilibrium (*c*_e_, mg dm^−3^) according to the following equation:

(6) $$K_{c}=\frac{c_{0}-c_{e}}{c_{e}}\;.$$

It is known that three adsorption thermodynamic parameters are related by the following equation [36]:

(7) $$\Delta G^{o}=\Delta H^{o}\;-\;T\;\cdot \;\Delta S^{o},$$

while the values of standard enthalpy and standard entropy changes can be calculated from van’t Hoff equation:

(8) $$ln\;(K_{c})=-\frac{\Delta H^{o}}{R}\;\cdot \;\frac{1}{T}+\frac{\Delta S^{o}}{R}.$$

**Figure 11:**
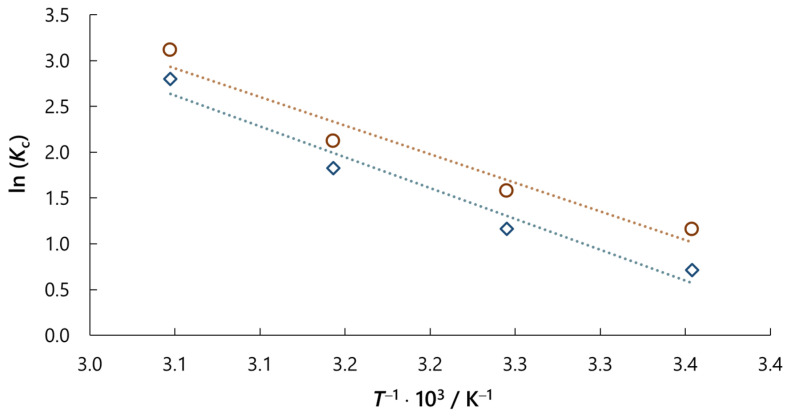
J. H. van’t Hoff plot for adsorption of RB5 dye on activated carbon with the salt addition in concentration of *c*_0_ = 0.01 M for NaCl (**◇**) and Na_2_SO_4_ (**◯**).

J. H. van’t Hoff plot of the ln(*K*_c_) vs. 1/*T* for NaCl and Na_2_SO_4_ concentration of *c*_0_ = 0.01 M resulted in a straight line with high correlation coefficients of 96.2% and 94.7%, respectively (Figure 11). Based on this plot, standard enthalpy change values (Δ*H*^o^) can be calculated from the slope, while standard entropy change values (Δ*S*^o^) from the intercept.

Table 4 summarises the thermodynamic parameters (Δ*G*^o^, Δ*H*^o^, Δ*S*^o^) derived from experimental data using Equations (5)–(8). The negative Δ*G*^o^ values confirm that RB5 dye adsorption onto activated carbon occurred spontaneously, requiring no external energy input. Additionally, the more negative Δ*G*° values with increasing temperature suggest greater thermodynamic favourability of adsorption at higher temperatures. This could be explained by the increased mobility of dye ions in solution at higher temperatures, which strengthens adsorbate–adsorbent affinity [37]. The positive Δ*H*° values reveal that the adsorption process for both salts was endothermic, in contrast to the typical exothermic nature of adsorption. This anomaly could be attributed to the desorption of water molecules initially bound to dye ions, followed by their subsequent adsorption onto activated carbon [36].

Based on the standard enthalpy change values, the adsorption mechanism can be proposed, as these values can obtain an insight into the type of bond between adsorbent and adsorbate. Generally, the enthalpy change values for chemisorption are between 40 and 120 kJ mol^−1^, which is much bigger than that for physisorption [38,39]. Therefore, values of ca. 56 and 52 kJ mol^−1^ for the adsorption of RB5 dye on activated carbon with the addition of salts are likely due to chemisorption. However, as determined in this study, intermolecular interactions, such as electrostatic interactions, hydrogen bonds, cation/anion–π and π–π interactions, also participate in the adsorption process. The positive value of Δ*S*^o^ suggests the affinity of activated carbon for RB5 dye and increased randomness at the interface between solid and liquid, i.e., between activated carbon and dye solution. During the adsorption process, the water-coordinated molecules are displaced by dye ions and consequently gain more translational entropy than is lost by the dye ions, thus allowing for the prevalence of randomness in the system. The positive Δ*S*^o^ value also corresponds to an increase in the degree of freedom of the adsorbed species [35,36,37,40].

## 3. Materials and Methods

### 3.1. Chemicals

Reactive Black 5 (RB5) dye was provided by Everlight Chemical Industrial Corp. (Taipei, Taiwan, C.I. 20505; chemical formula: C_26_H_21_N_5_Na_4_O_19_S_6_; molecular weight: 991.82; purity: 75–80%, *w*/*w*). The chemical structure of the dye (Figure 1) was drawn by the Biovia Draw program, version 21.1. The adsorbent used was powdered activated carbon, supplied by Kemika company, Zagreb, Croatia, with particle sizes of <40 μm (85%) and >80 μm (5%). It was dried for 24 h in an oven at 105 °C and stored in a desiccator until it was used.

### 3.2. Adsorbent Characterisation

The specific surface area of activated carbon was determined by the B.E.T. technique by N_2_ adsorption isotherms using the Gemini 2380 instrument (Micromeritics, Norcross, GA, USA).

Fourier transform infrared (FTIR) spectra of dried samples were recorded on the TENSOR II (Bruker, Billerica, MA, USA) in the 340–4000 cm^−1^ region using the KBr pellet technique. The samples were prepared for the analysis by mixing a dry KBr (IR grade) with a small amount of sample.

The zeta potential of the activated carbon was measured using a Zetasizer Nano ZS (Malvern, UK). NaCl and HCl solutions, i.e., Na_2_SO_4_ and H_2_SO_4_ solutions (*c* = 10^–3^ mol dm^–3^), were added into the initial suspension of activated carbon (*γ* = 0.1 g dm^–3^) in deionised water to reach pH ≈ 3. In order to obtain an isoelectric point, the suspension was titrated with 0.1 mol dm^–3^ NaOH solution from pH ≈ 3 to pH ≈ 9 using the MPT-3 Multi-Purpose Titrator (Malvern). Measurements were performed in triplicates at 25 °C, and the lines in Figure 4 represent the average values of the three points obtained. From electrophoretic mobilities, the electrokinetic ζ-potential was calculated using the Smoluchowski equation.

A field emission scanning electron microscope (Mira, Tescan, Brno, Czech) was used to visualise the adsorbent’s morphology. The accelerating voltage was 10.00 kV, and the scanning was performed in situ on a sample powder. Samples were previously coated with gold/palladium in a sputter coater. Optical micrographs were recorded with a Nikon Elipse E 400 microscope (Tokyo, Japan).

### 3.3. Batch Mode Adsorption Studies

A solution of RB5 dye of the concentration of *c*_0_ = 500 mg dm^−3^ was prepared by dissolving the dye in a small amount of deionised water. Subsequently, the appropriate amount of salt was dissolved in deionised water and mixed with the RB5 dye solution in a volumetric flask. The mixture was then diluted with deionised water to achieve the appropriate volume. Four dye solutions with different NaCl concentrations (*c*_0_ = 0.01, 0.05, 0.10 and 1.00 mol dm^−3^) and one with Na_2_SO_4_ (*c*_0_ = 0.01 mol dm^−3^) were prepared in order to examine the effect of ionic strength on the adsorption process. Adsorption studies involved contacting 50 cm^3^ of the RB5 dye solution containing salt with 0.1 g (±0.1 mg) of powdered activated carbon in glass bottles. The suspensions were agitated at different contact times (15, 30, 45, 60, 120 min and 16 h until equilibrium was reached) with an impeller speed of 250 rpm at a temperature of 45(±1) °C using a Heidolph Unimax 1010 with Incubator 1000 (Schwabach, Germany). Our previous studies established that 16 h was sufficient to reach adsorption equilibrium [19,20]. Experiments with NaCl and Na_2_SO_4_ (*c*_0_ = 0.01 mol dm^−3^) added to the RB5 dye solution were additionally conducted at temperatures of 25(±1), 35(±1) and 55(±1) °C in order to investigate the influence of temperature on the adsorption process, a well as to determine thermodynamic parameters of adsorption. After agitation, the suspensions were filtered through blue ribbon filter paper, and the residual dye concentration in the liquid phase was determined spectrophotometrically using a UV–Vis spectrophotometer Perkin Elmer Lambda 20 at the maximum absorbance wavelength (*λ*_max_ = 598 nm). The calibration graph of absorbance vs. concentration followed a linear Beer–Lambert relationship. To assess whether the addition of salts affected the pH value and, consequently, the adsorption, pH measurements were taken for all filtrates after the experiments using a ProLab 3000 pH meter (SI Analytics, Mainz, Germany). All experiments were repeated three times under identical conditions to ensure repeatability, and the points presented in figures and experimental data in tables represent the average values of these three repetitions.

The amount of adsorbed dye at time *t* and at equilibrium (*q*_t_ and *q*_e_, mg g^−1^) were calculated by using the following equation:

(9) $$q=\frac{V\;\cdot \;\left(c_{0}-c_{t}\right)}{m},$$

where *c*_0_ is the initial dye concentration (*c*_0_ = 500 mg dm^−3^), *c*_t_ is its concentration in the liquid phase at time *t* and at equilibrium (*t* = 16 h), *V* is the volume of liquid phase (dm^3^), and *m* is mass of the adsorbent (g).

The efficiency of dye adsorption, i.e., the efficiency of decolouration (*E*_d_), is calculated by the following equation:

(10) $$E_{d}=\frac{\left(c_{0}-c_{t}\right)}{c_{0}}\times 100.$$

## 4. Conclusions

In this research, we investigated the influence of ionic strength on the adsorption of Reactive Black 5 dye on commercial activated carbon. Besides sodium chloride, sodium sulphate was used for the first time in the adsorption of RB5 dye on activated carbon. It was proved that higher ionic strength, either usage of higher NaCl concentration or Na_2_SO_4_ instead of NaCl of the same concentration, resulted in higher removal of Reactive Black 5 dye from the solution. Even the addition of NaCl in the concentration of *c*_0_ = 0.01 M significantly increased the efficiency of decolouration, while the highest adsorption was observed for *c*_0_ (NaCl) = 1.00 M. In addition, higher temperatures reinforced adsorption regardless of the salt used in the adsorption experiment. The results of zeta-potential and FTIR spectra analysis showed that the adsorption of RB5 dye on activated carbon was governed by various intermolecular interactions (electrostatic interactions, hydrogen bonds, and cation/anion–π and π–π interactions). This research also demonstrated that the adsorption of RB5 dye on activated carbon followed a pseudo-second-order kinetic model and that intraparticle diffusion is not the only process that influences the adsorption rate. Furthermore, the negative standard Gibbs free energy change values indicated that the adsorption process occurred spontaneously and that higher temperatures favour adsorption. Finally, the positive standard enthalpy change values revealed that the adsorption was an endothermic process, whereas the positive standard entropy change values suggested that the solid–liquid interface became increasingly disordered during the adsorption process.

In conclusion, this study showed that salts present in coloured effluents after the dyeing process could dramatically increase dye adsorption on commercially available activated carbon. The salts used in this research, NaCl and Na_2_SO_4_, are usually added to dyeing baths, and the dyeing process is performed at higher temperatures. Therefore, these conditions could be employed for more efficient removal of dyes by adsorption after the dyeing process.

## Figures and Tables

**Table 1:** Amounts of adsorbed RB5 dye (*q*_t_) after an appropriate time of adsorption (*t*) at 45 °C without salt addition [23] and for all initial NaCl concentrations (*c*_0_ = 0.01, 0.05, 0.10 and 1.00 M).

*t*/ min	*q*_t_/mg g^−1^				
	*c* (NaCl)				
	Data from [23]	0.01 M	0.05 M	0.10 M	1.00 M
15	63.5	100.3	149.9	167.9	214.3
30	83.6	126.9	166.0	185.2	233.4
45	95.7	134.5	179.8	193.9	237.4
60	100.7	138.4	183.5	200.7	240.4
120	132.1	156.5	203.1	219.4	247.5
960	190.9	215.4	243.4	248.0	249.9

**Table 2:** The efficiency of decolouration (*E*_d_) after appropriate time of adsorption (*t*) at 45 °C without salt addition [23] and for the NaCl and Na_2_SO_4_ concentration of *c*_0_ = 0.01 M.

*t*/ min	*E*_d_/%		
	Data from [23]	NaCl	Na_2_SO_4_
15	25.4	40.1	42.7
30	33.4	50.8	51.1
45	38.3	53.8	57.8
60	40.3	55.4	59.8
120	52.8	62.6	68.7
960	76.3	86.2	89.3

**Table 3:** Kinetic parameters for RB5 dye adsorption on activated carbon at 45 °C for all NaCl initial concentrations (*c*_0_ = 0.01–1.00 M) and Na_2_SO_4_ concentration of *c*_0_ = 0.01 M.

Salt	*c*_0_/ mol dm^−3^	*q*_e,exp._/ mg g^−1^	Pseudo-First-Order Model			Pseudo-Second-Order Model			
			*q*_e,calc._/ mg g^−1^	*R* ^2^	*k*_1_/ min^−1^	*q*_e,calc._/ mg g^−1^	*R* ^2^	*k*_2_/ g mg^−1^ min^−1^	*h*/ mg g^−1^ min^−1^
NaCl	0.01	215.4	113.8	0.8961	0.0058	222.2	0.9991	1.46 × 10^−4^	7.21
	0.05	243.4	97.0	0.9644	0.0076	250.0	0.9998	2.25 × 10^−4^	14.09
	0.10	248.0	85.8	0.9841	0.0094	250.0	0.9999	3.18 × 10^−4^	19.88
	1.00	249.9	40.6	0.9748	0.0240	250.0	1.0000	1.78 × 10^−3^	111.1
Na_2_SO_4_	0.01	223.2	119.3	0.9519	0.0073	227.3	0.9996	1.60 × 10^−4^	8.29

**Table 4:** Thermodynamic parameters for the adsorption of RB5 dye on activated carbon with the addition of NaCl and Na_2_SO_4_ (*c*_0_ = 0.01 M).

Salt	*T*/ K	*c*_e_/ mg dm^−3^	*c*_0_ − *c*_e_/ mg dm^−3^	*K*_c_	∆*G*°/ kJ mol^−1^	∆*H*°/ kJ mol^−1^	∆*S*°/ J mol^−1^ K^−1^
NaCl	298	164.3	335.7	2.0432	−1.77	55.97	192.48
	308	119.1	380.9	3.1982	−2.98		
	318	69.2	430.8	6.2254	−4.84		
	328	28.7	471.3	16.4216	−7.64		
Na_2_SO_4_	298	119.6	380.4	3.1806	−2.87	51.90	182.51
	308	85.4	414.6	4.8548	−4.05		
	318	53.6	446.4	8.3284	−5.61		
	328	21.2	478.8	22.5849	−8.50		

## Data Availability

Data not contained within the article or Supplementary Material are available on request from the authors.

## Supplementary Materials

The following supporting information can be downloaded at: https://www.mdpi.com/article/10.3390/molecules30122593/s1, Table S1: Efficiency of decolouration (*E*_d_) after appropriate time of adsorption (*t*) at 45 °C for all initial NaCl concentrations (*c*_0_ = 0.01, 0.05, 0.10 and 1.00 M) and Na_2_SO_4_ concentration of *c*_0_ = 0.01 M; Table S2: Efficiency of decolouration (*E*_d_) after appropriate time of adsorption (*t*) for NaCl and Na_2_SO_4_ concentration of *c*_0_ = 0.01 M at 25, 35, 45 and 55 °C; Table S3: pH values for RB5 dye adsorption after appropriate time of adsorption (*t*) for NaCl and Na_2_SO_4_ concentration of *c*_0_ = 0.01 M at 25, 35 and 55 °C; Table S4: Kinetic parameters for RB5 dye adsorption on activated carbon for NaCl and Na_2_SO_4_ concentration of *c*_0_ = 0.01 M at 25, 35 and 55 °C; Figure S1: FTIR spectrum of commercial powdered activated carbon; Figure S2: Amounts of adsorbed dye (*q*_t_) after appropriate time of adsorption (*t*) at 45 °C for all NaCl concentrations and without salt addition during adsorption process [23]; Figure S3: pH profiles for RB5 dye adsorption after appropriate time of adsorption (*t*) at 45 °C for all NaCl concentrations and Na_2_SO_4_ concentration of *c*_0_ = 0.01 M; Figure S4: Graphical representation of linear form of pseudo-first-order (a) and pseudo-second-order (b) kinetic models for adsorption of RB5 dye on activated carbon at 45 °C for all NaCl concentrations and Na_2_SO_4_ concentration of *c*_0_ = 0.01 M; Figure S5: Graphical representation of linear form of pseudo-first-order (a) and pseudo-second-order (b) kinetic models for adsorption of RB5 dye on activated carbon for NaCl concentration of c_0_ = 0.01 M at 25, 35 and 55 °C; Figure S6: Graphical representation of linear form of pseudo-first-order (a) and pseudo-second-order (b) kinetic models for adsorption of RB5 dye on activated carbon for Na_2_SO_4_ concentration of c_0_ = 0.01 M at 25, 35 and 55 °C; Figure S7: Root time plot for the adsorption of RB5 dye on activated carbon for NaCl concentration of *c*_0_ = 0.01 M at 25, 35 and 55 °C; Figure S8: Root time plot for the adsorption of RB5 dye on activated carbon for Na_2_SO_4_ concentration of *c*_0_ = 0.01 M at 25, 35 and 55 °C.
