# Supplementary material for: Influence of Ionic Strength and Temperature on the Adsorption of Reactive Black 5 Dye by Activated Carbon: Kinetics, Mechanisms and Thermodynamics
Source: Molecules. 2025 Jun 14;30(12):2593. doi: 10.3390/molecules30122593 (PMC12195980; doi:10.3390/molecules30122593)
Supplement: Supplementary file 1 [file molecules-30-02593-s001.zip › molecules-3549217-supplementary.pdf]

Influence of Ionic Strength and Temperature on the Adsorption of Reactive Black 5 Dye by Activated Carbon: Kinetics, Mechanisms and Thermodynamics

Mario Cetina *, Petra Mihovilović, Ana Pešić and Branka Vojnović

Department of Applied Chemistry, University of Zagreb Faculty of Textile Technology, Prilaz Baruna Filipovića 28a, HR-10000 Zagreb, Croatia
* Correspondence: mario.cetina@ttf.unizg.hr

SUPPLEMENTARY MATERIAL

<u>List of tables and figures</u>:

Table S1. Efficiency of decolouration (E_d) after appropriate time of adsorption (t) at 45°C for all initial NaCl concentrations (c_0 = 0.01, 0.05, 0.10 and 1.00 M) and Na_2SO_4 concentration of c_0 = 0.01 M

Table S2. Efficiency of decolouration (E_d) after appropriate time of adsorption (t) for NaCl and Na_2SO_4 concentration of c_0 = 0.01 M at 25, 35, 45 and 55°C

Table S3. pH values for RB5 dye adsorption after appropriate time of adsorption (t) for NaCl and Na_2SO_4 concentration of c_0 = 0.01 M at 25, 35 and 55°C

Table S4. Kinetic parameters for RB5 dye adsorption on activated carbon for NaCl and Na_2SO_4 concentration of c_0 = 0.01 M at 25, 35 and 55°C

Figure S1. FTIR spectrum of commercial powdered activated carbon

Figure S2. Amounts of adsorbed dye (q_t) after appropriate time of adsorption (t) at 45°C for all NaCl concentrations (○ c_0 = 0.01 M; ◇ c_0 = 0.05 M; Δ c_0 = 0.10 M; □ c_0 = 1.00 M) and without salt addition during adsorption process [23] (✱)

Figure S3. pH profiles for RB5 dye adsorption after appropriate time of adsorption (t) at 45°C for all NaCl concentrations (○ c_0 = 0.01 M; ◇ c_0 = 0.05 M; Δ c_0 = 0.10 M; × c_0 = 1.00 M) and Na_2SO_4 concentration of c_0 = 0.01 M (□)

Figure S4. Graphical representation of linear form of pseudo-first-order (a) and pseudo-second-order (b) kinetic models for adsorption of RB5 dye on activated carbon at 45°C for all NaCl concentrations (○ c_0 = 0.01 M; ◇ c_0 = 0.05 M; Δ c_0 = 0.10 M; □ c_0 = 1.00 M) and Na_2SO_4 concentration of c_0 = 0.01 M (✱)

Figure S5. Graphical representation of linear form of pseudo-first-order (a) and pseudo-second-order (b) kinetic models for adsorption of RB5 dye on activated carbon for NaCl concentration of c_0 = 0.01 M at 25, 35 and 55°C (○ 25°C; ◇ 35°C; Δ 55°C)

Figure S6. Graphical representation of linear form of pseudo-first-order (a) and pseudo-second-order (b) kinetic models for adsorption of RB5 dye on activated carbon for Na_2SO_4 concentration of c_0 = 0.01 M at 25, 35 and 55°C (○ 25°C; ◇ 35°C; Δ 55°C)

Figure S7. Root time plot for the adsorption of RB5 dye on activated carbon for NaCl concentration of c_0 = 0.01 M at 25, 35 and 55°C (◇ 25°C; □ 35°C; ○ 55°C)

Figure S8. Root time plot for the adsorption of RB5 dye on activated carbon for Na_2SO_4 concentration of c_0 = 0.01 M at 25, 35 and 55°C (◇ 25°C; □ 35°C; ○ 55°C)

Table S1. Efficiency of decolouration (E_d) after appropriate time of adsorption (t) at 45°C for all initial NaCl concentrations (c_0 = 0.01, 0.05, 0.10 and 1.00 M) and Na_2SO_4 concentration of c_0 = 0.01 M

***t* / min**	***E*d / %**				
	**NaCl**				**Na_2SO_4**
	0.01 M	0.05 M	0.10 M	1.00 M	0.01 M
15	40.1	59.9	67.2	85.7	42.7
30	50.8	66.4	74.1	93.4	51.1
45	53.8	71.9	77.6	94.9	57.8
60	55.4	73.4	80.3	96.2	59.8
120	62.6	81.2	87.8	99.0	68.7
960	86.2	97.3	99.2	99.9	89.3

Table S2. Efficiency of decolouration (E_d) after appropriate time of adsorption (t) for NaCl and Na_2SO_4 concentration of c_0 = 0.01 M at 25, 35, 45 and 55°C

***t* / min**	***E*d / %**							
	**25°C**		**35°C**		**45°C** [1]		**55°C**	
	NaCl	Na_2SO_4	NaCl	Na_2SO_4	NaCl	Na_2SO_4	NaCl	Na_2SO_4
15	36.7	43.2	40.0	45.2	40.1	42.7	46.9	50.1
30	41.8	48.2	46.5	52.7	50.8	51.1	55.9	57.3
45	43.8	51.3	49.3	55.9	53.8	57.8	60.4	64.5
60	45.9	51.9	51.7	57.6	55.4	59.8	65.5	67.6
120	50.8	57.1	56.1	62.9	62.6	68.7	71.6	76.3
960	67.1	76.1	76.2	82.9	86.2	89.3	94.3	95.8

[1] although presented in Table 2, data for 45°C are also given for comparison

Table S3. pH values for RB5 dye adsorption after appropriate time of adsorption (*t*) for NaCl and Na_2SO_4 concentration of c_0 = 0.01 M at 25, 35 and 55°C

**t / min**	**pH**					
	**25°C**		**35°C**		**55°C**	
	NaCl	Na_2SO_4	NaCl	Na_2SO_4	NaCl	Na_2SO_4
15	6.84	6.89	6.98	6.94	5.71	6.96
30	7.04	6.86	7.05	6.96	6.98	7.01
45	6.95	6.98	7.09	6.99	7.02	7.05
60	7.08	6.93	7.09	6.94	6.95	7.01
120	7.14	7.01	7.11	7.03	7.06	6.91
960	7.14	7.17	7.09	7.03	6.84	6.54

Table S4. Kinetic parameters for RB5 dye adsorption on activated carbon for NaCl and Na_2SO_4 concentration of c_0 = 0.01 M at 25, 35 and 55°C

**Salt**	**t / °C**	**$q_{e,exp.}$ / mg g^{-1}**	**pseudo–first-order model**			**pseudo–second-order model**			
			**$q_{e,calc.}$ / mg g^{-1}**	**R^2**	**k_1 / min^{-1}**	**$q_{e,calc.}$ / mg g^{-1}**	**R^2**	**k_2 / g mg^{-1}min^{-1}**	**h / mg g^{-1} min^{-1}**
NaCl	25	167.8	76.9	0.9544	0.0055	172.4	0.9993	$2.25\cdot10^{-4}$	6.69
	35	190.4	88.8	0.9036	0.0051	196.1	0.9991	$1.86\cdot10^{-4}$	7.14
	55	235.6	118.7	0.9216	0.0066	243.9	0.9994	$1.52\cdot10^{-4}$	9.03
Na_2SO_4	25	190.2	82.0	0.9291	0.0048	196.1	0.9991	$2.02\cdot10^{-4}$	7.76
	35	207.3	92.2	0.9039	0.0055	212.8	0.9993	$1.90\cdot10^{-4}$	8.61
	55	239.4	119.7	0.9634	0.0079	243.9	0.9997	$1.73\cdot10^{-4}$	10.31

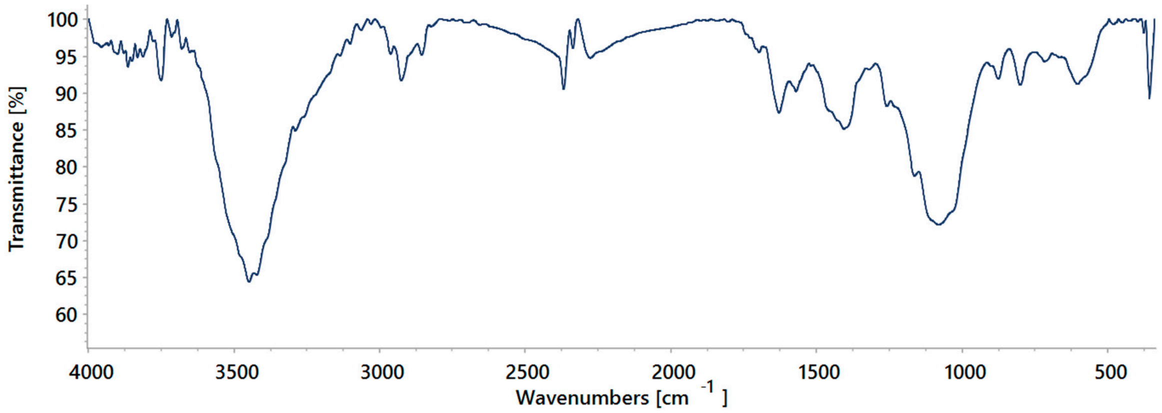

Figure S1. FTIR spectrum of commercial powdered activated carbon

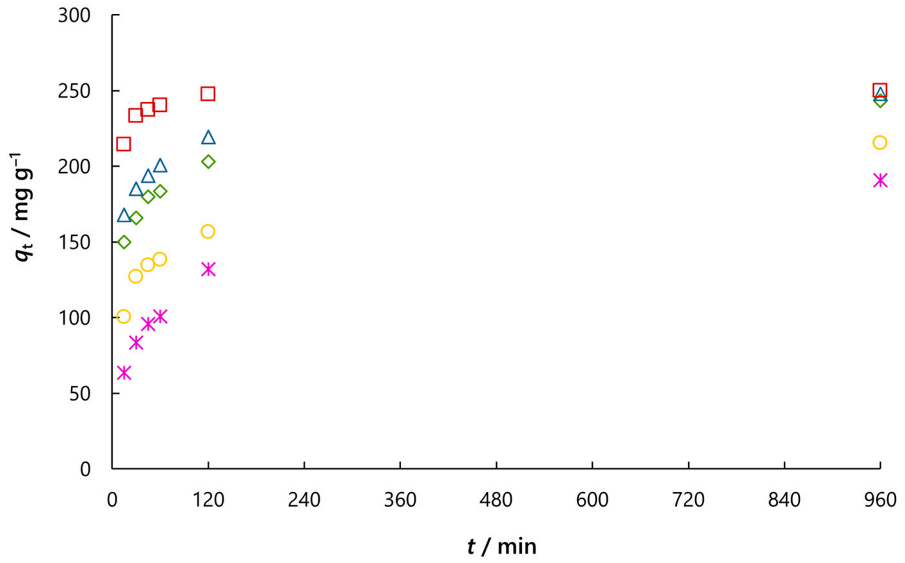

Figure S2. Amounts of adsorbed dye (q_t) after appropriate time of adsorption (t) at 45°C for all NaCl concentrations (○ c_0 = 0.01 M; ◇ c_0 = 0.05 M; Δ c_0 = 0.10 M; □ c_0 = 1.00 M) and without salt addition during adsorption process [23] (✱)

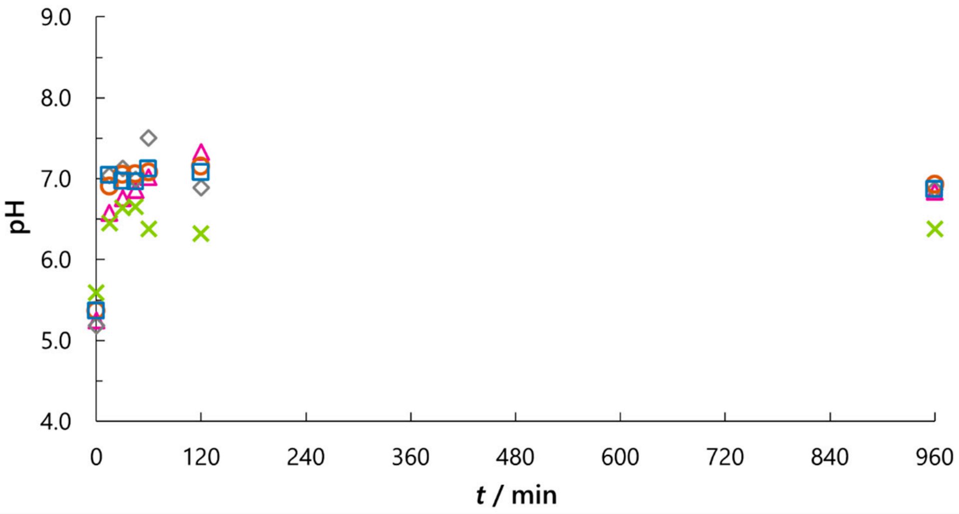

Figure S3. pH profiles for RB5 dye adsorption after appropriate time of adsorption (t) at 45°C for all NaCl concentrations (○ c_0 = 0.01 M; ◇ c_0 = 0.05 M; Δ c_0 = 0.10 M; × c_0 = 1.00 M) and Na_2SO_4 concentration of c_0 = 0.01 M (□)

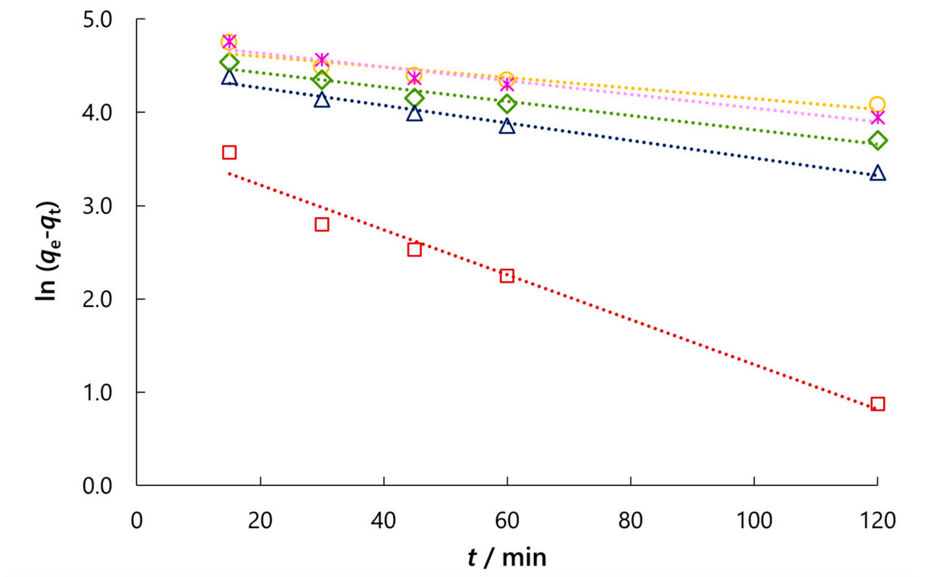

(a)

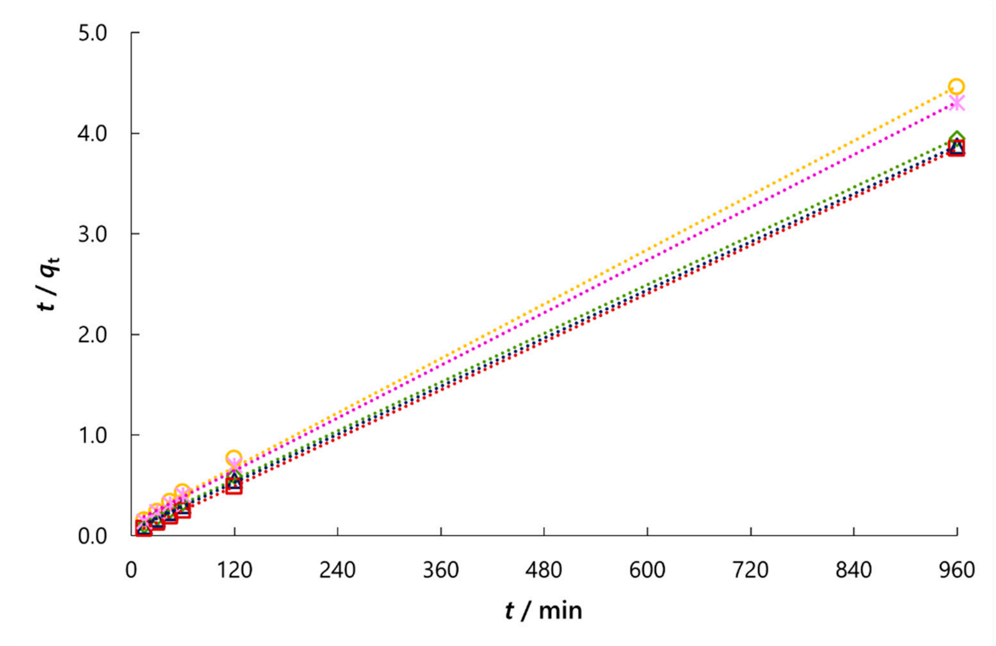

(b)

Figure S4. Graphical representation of linear form of pseudo-first-order (a) and pseudo-second-order (b) kinetic models for adsorption of RB5 dye on activated carbon at 45°C for all NaCl concentrations (○ c_0 = 0.01 M; ◇ c_0 = 0.05 M; **Δ** c_0 = 0.10 M; □ c_0 = 1.00 M) and Na_2SO_4 concentration of c_0 = 0.01 M (✱)

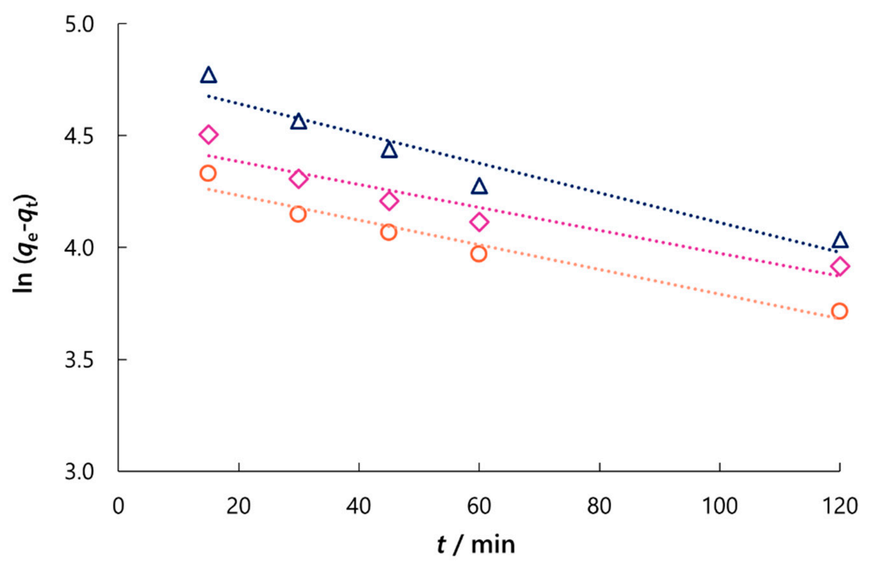

(a)

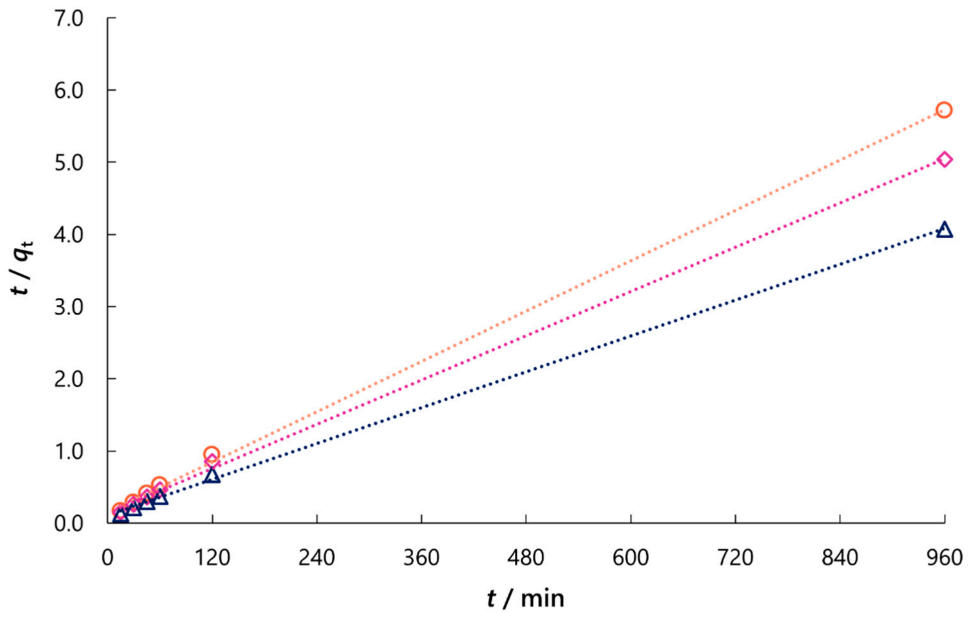

(b)

Figure S5. Graphical representation of linear form of pseudo-first-order (a) and pseudo-second-order (b) kinetic models for adsorption of RB5 dye on activated carbon for NaCl concentration of c_0 = 0.01 M at 25, 35 and 55°C (○ 25°C; ◇ 35°C; Δ 55°C)

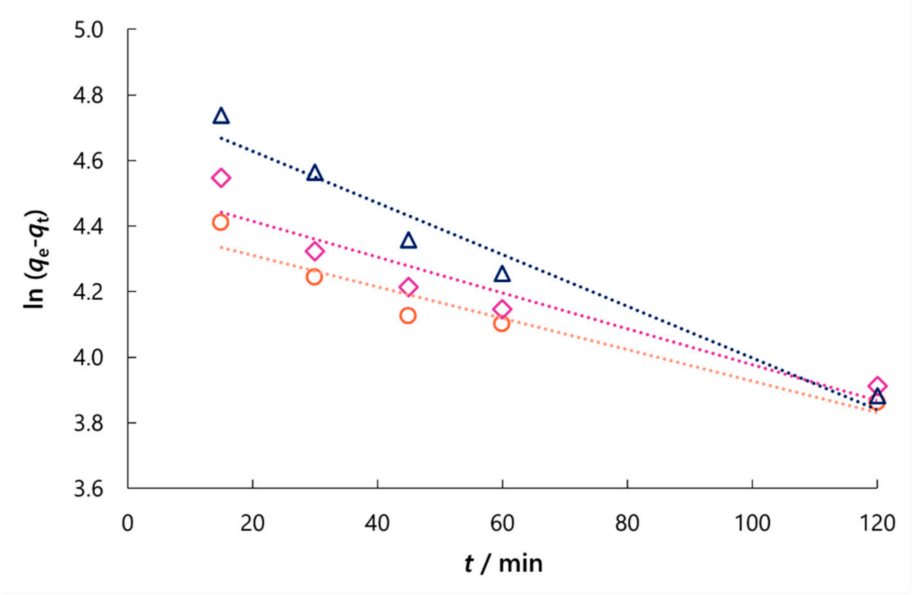

(a)

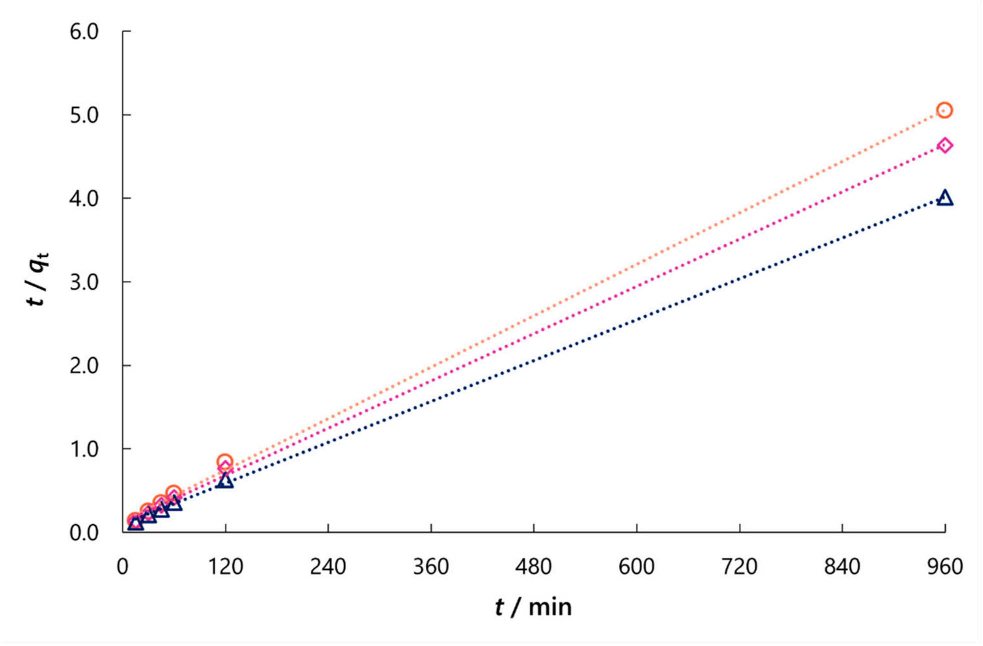

(b)

Figure S6. Graphical representation of linear form of pseudo-first-order (a) pseudo-second-order (b) kinetic models for adsorption of RB5 dye on activated carbon for Na_2SO_4 concentration of c_0 = 0.01 M at 25, 35 and 55°C (○ 25°C; ◇ 35°C; Δ 55°C)

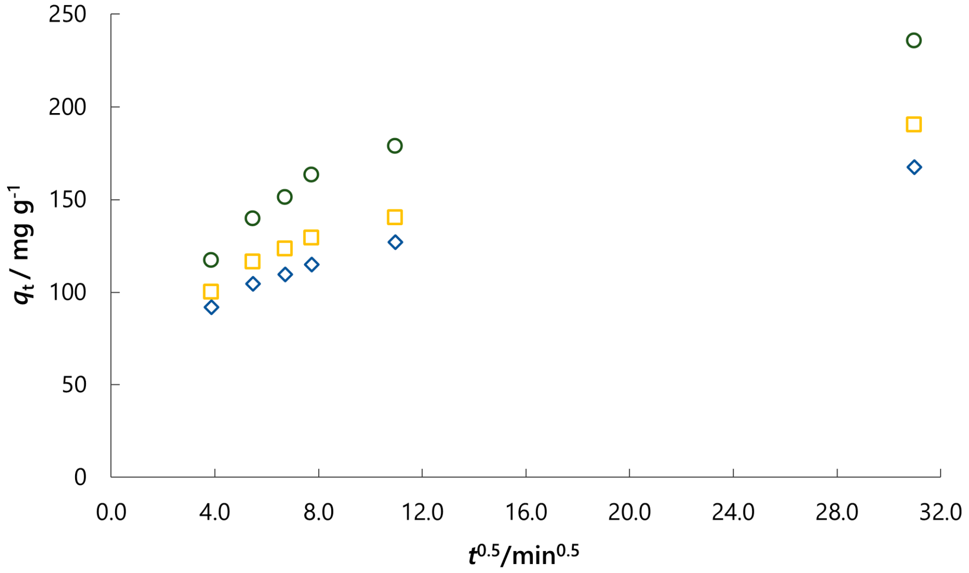

Figure S7. Root time plot for the adsorption of RB5 dye on activated carbon for NaCl concentration of c_0 = 0.01 M at 25, 35 and 55°C (◇ 25°C; □ 35°C; **O** 55°C)

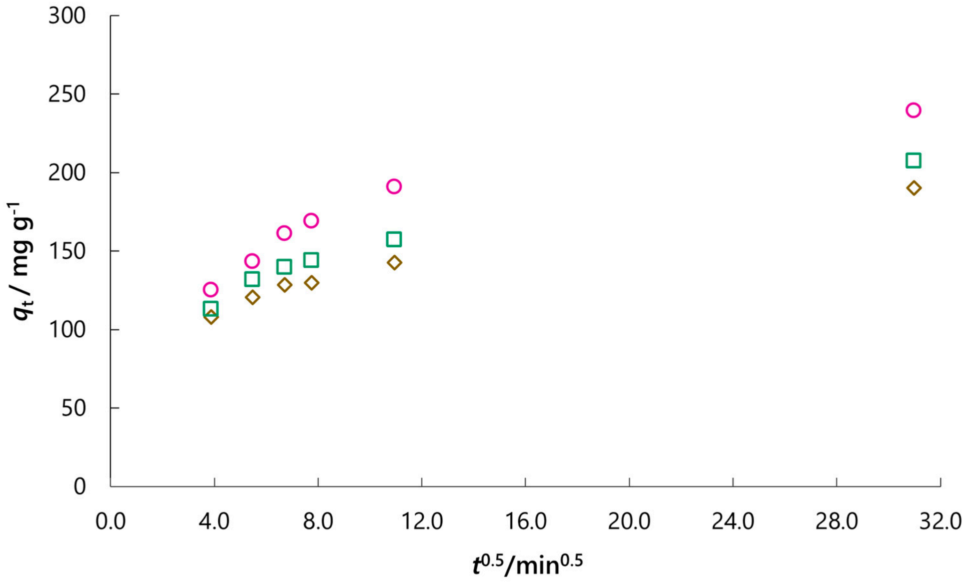

Figure S8. Root time plot for the adsorption of RB5 dye on activated carbon for Na_2SO_4 concentration of c_0 = 0.01 M at 25, 35 and 55°C (◇ 25°C; □ 35°C; **O** 55°C)
